# Identifying non-specific low back pain clinical subgroups from sitting and standing repositioning posture tasks using a novel Cardiff Dempster–Shafer Theory Classifier

**DOI:** 10.1016/j.clinbiomech.2019.10.004

**Published:** 2019-12

**Authors:** Liba Sheeran, Valerie Sparkes, Gemma Whatling, Paul Biggs, Cathy Holt

**Affiliations:** a School of Healthcare Sciences, Cardiff University, Cardiff, Wales, United Kingdom; b Cardiff School of Engineering, Cardiff University, Cardiff, Wales, United Kingdom; c Biomechanics and Bioengineering Research Centre Versus Arthritis, Cardiff University, Cardiff, Wales, United Kingdom

**Keywords:** Non-specific low back pain, Dempster-Shaffer Theory Classifier, Objective classification

## Abstract

**Background:**

Low back pain (LBP) classification systems are used to deliver targeted treatments matched to an individual profile, however, distinguishing between different subsets of LBP remains a clinical challenge.

**Methods:**

A novel application of the Cardiff Dempster–Shafer Theory Classifier was employed to identify clinical subgroups of LBP on the basis of repositioning accuracy for subjects performing a sitting and standing posture task. 87 LBP subjects, clinically subclassified into flexion (n = 50), passive extension (n = 14), and active extension (n = 23) motor control impairment subgroups and 31 subjects with no LBP were recruited. Thoracic, lumbar and pelvic repositioning errors were quantified. The Classifier then transformed the error variables from each subject into a set of three belief values: (i) consistent with no LBP, (ii) consistent with LBP, (iii) indicating either LBP or no LBP.

**Findings:**

In discriminating LBP from no LBP the Classifier accuracy was 96.61%. From no-LBP, subsets of flexion LBP, active extension and passive extension achieved 93.83, 98.15% and 97.62% accuracy, respectively. Classification accuracies of 96.8%, 87.7% and 70.27% were found when discriminating flexion from passive extension, flexion from active extension and active from passive extension subsets, respectively. Sitting lumbar error magnitude best discriminated LBP from no LBP (92.4% accuracy) and the flexion subset from no-LBP (90.1% accuracy). Standing lumbar error best discriminated active and passive extension from no LBP (94.4% and 95.2% accuracy, respectively).

**Interpretation:**

Using repositioning accuracy, the Cardiff Dempster–Shafer Theory Classifier distinguishes between subsets of LBP and could assist decision making for targeted exercise in LBP management.

## Highlights

• Objective classifier discriminated back pain subsets based on repositioning sense.
• An accuracy comparable to that of expert clinician agreement was achieved.
• Sitting repositioning error best discriminated flexion related back pain.
• Standing repositioning error best discriminated extension related back pain.

## 1 Introduction

Low back pain (LBP) is the greatest global contributor to long term disability (Hoy et al., 2014). The lifetime incidence of LBP is 58–84% (Manchikanti et al., 2014). Whilst majority of LBP resolves within a 4–6 weeks, up to 33% of people have a recurrence within 1 year (Silva et al., 2017). The majority of LBP is non-specific with no identifiable pathoanatomical cause (Maher et al., 2017). Non-specific LBP (NSLBP) is characterised by pain and reduced physical function, associated with significant decline in mental and physical health, increased risk of developing chronic health conditions and all-cause mortality (Briggs et al., 2016). NICE guidelines endorse active physical interventions, including exercise and keeping physically active, as the treatment of choice (NICE, 2017).

Adhering to these clinical guidelines is challenging however, as people with NSLBP exhibit demonstrable physical impairments including reduced range of motion, flexibility (Sadler et al., 2017), proprioception deficits (Willigenburg et al., 2013) and altered muscle function (Hodges, 2011). In addition, these physical impairments have been shown to be highly variable (Astfalck et al., 2010; Dankaerts et al., 2006b; Hemming et al., 2017; Sheeran et al., 2012), posing a challenge in exercise therapy design.

Clinical classification models such as the multi-dimensional classification system (MDCS) (O'Sullivan, 2006), that subgroup NSLBP into distinct subsets on the basis of posture and movement pain behaviour, can be utilised to help in clinical decision making when individualising exercise programmes. These clinical subsets have demonstrated consistent, distinct differences in spinal and pelvic kinematics during sitting and standing (Astfalck et al., 2010; Dankaerts et al., 2006b; Sheeran et al., 2012). MDCS-based therapies have been also found to produce superior improvements in pain and disability compared to standard practice in NSLBP patients (Fersum et al., 2012; Sheeran et al., 2013). Utilising classification processes in clinical practice however, is considered challenging given the complexity of synthesizing a large amount of clinical information requiring many hours of advanced training (Sheeran et al., 2015).

There is a growing body of research describing the use of objective multivariate classification methods that combine a wealth of corroborating and conflicting clinical and biomechanical evidence to assist clinical decision making (Beynon et al., 2006; Jones et al., 2006; Whatling et al., 2008). A range of complex multivariate statistical methods such as principal component analysis (PCA) and linear discriminative analysis (LDA) or cluster analysis (CA) techniques are increasingly used to analyse human movement (Boser et al., 2018; Clark et al., 2017; Witte et al., 2010). A novel Cardiff Dempster-Shafer Theory (DST) Classifier is an easy-to-operate method that is able to produce discriminatory models of function in healthy and pathological populations from objective analysis of clinical and biomechanical data (Beynon et al., 2006; Jones et al., 2006; Metcalfe et al., 2017; Whatling et al., 2008). With growing access to biomechanical data in NSLBP subgroups there is an opportunity to apply these powerful statistical techniques to distinguish objectively between function of individuals belonging to different NSLBP subsets. This could facilitate improved targeted exercise therapies as well as providing tools to monitor effectiveness in addressing movement aberrations in the spine.

The aim of this study was to assess whether the Cardiff DST Classifier method can distinguish between the three commonly encountered clinical MDCS subgroups of NSLBP (i) flexion pattern (FP) (ii) active extension pattern (AEP) and (iii) passive extension pattern (PEP) on basis of kinematic evaluation of repositioning sense in the lumbar and thoracic spine and pelvis during sitting and standing tasks. The secondary aim was to establish the top three parameters that most accurately characterise each LBP subtype. This is the first study to date to apply a DST Classifier to distinguish between different types of a single musculoskeletal condition, rather than purely classifying pathology from healthy function. It is anticipated that this new approach may simplify the process of classification aiding in targeting therapies for NSLBP that could be of significant clinical value to practitioners and patients.

## 2 Methods

### 2.1 Participants

The low back pain (LBP) group included individuals recruited from a physiotherapy waiting list at Cardiff and Vale University Health Board, Wales, U.K. Using the selection criteria in Table 1, individuals with LBP >12 weeks, with no leg pain were included. The pain-free (no-LBP) group was matched for age and gender with no spine or pelvic pain for 1 year minimum. The exclusion criteria were any vestibular, visual, or neurological conditions affecting balance. Ethical approval was obtained from South East Wales Research Ethics Committee (REC reference: WSE02/118) and Cardiff and Vale NHS Trust Research and development Committee (project ID: CLC/3789) with written consent gained from all participants. The LBP group had received no physiotherapy prior to the data collection or any other exercise intervention likely to influence posture task performance.

**Table 1:** LBP group selection criteria.

*Inclusion criteria*
Non-specific low back pain (LBP) > 12 weeks
Pain located between Thoracic level 12 and buttock line
Clear mechanical basis for the disorder (i.e. LBP eased and provoked by specific postures and movements as determined by subjective and objective clinical examination)

*Exclusion criteria*
Radicular pain with leg pain referral
Systemic inflammatory disease (e.g. psoriatic arthritis, rheumatoid arthritis)
Serious pathology (neurological conditions, diabetic neuropathy, cancer)
Psychological distress evidenced by Distress Risk Assessment Method (DRAM)
Pregnancy/breast feeding
Spinal surgery
Vestibular/visual/neurological dysfunction affecting sensory function
Unable to sit and/or stand unaided

### 2.2 Clinical classification

The LBP group was classified using a validated and reliable multi-dimensional classification system (MDCS) (Fersum et al., 2009). The classification in this study was conducted by two experienced physiotherapists (LS, VS) both fully trained in MDCS following a process detailed elsewhere (Fersum et al., 2009). In summary, this involved subjective assessment of pain behaviour, review of radiological imaging, pain cognitions and beliefs, lifestyle behaviours and objective assessment of spinal function (forward/backward bend, sit-to-stand, standing, slumped, usual, upright sitting posture, single leg stand). A clinical judgement is then made as to whether any observed movement aberration is adaptive (i.e. protective) or mal-adaptive (i.e. pain driver) and individuals are broadly categorised into movement directional sub-types. LBP individuals classified by both physiotherapists (LS, VS) with flexion pattern (FP), active extension pattern (AEP) and passive extension pattern (PEP) were selected for this study on the basis of being the most frequently encountered clinically (Fersum et al., 2009). Clinical features of these LBP subsets are in Table 2.

**Table 2:** Clinical features of flexion pattern (FP), active extension pattern (AEP) and passive extension pattern (PEP).

Pattern	Features
*FP*	Flat lumbar spine with loss of lumbar curvature (hypolordosis)
	Pain exacerbated by movements/postures involving excessive lumbar flexion
	Pain provoking activities tend to include flexion-based postures/movements (e.g. prolonged sitting, bending, driving, lifting)
	Pain easing activities tend to include extension-based postures/movements (bending spine backwards)
*AEP*	Actively adopted extended lumbar spine with excessive lumbar curvature (hyperlordosis)
	Pain exacerbated by movement/postures involving active lumbar extension
	Pain provoking activities tend to include extension-based postures/movements (e.g. prolonged standing, walking, supported lordosis sitting)
	Pain eased by movement/postures involving lumbar flexion (e.g. bending forwards)
*PEP*	Passively adopted extended lumbar spine with excessive lumbar curvature (hyperlordosis) resulting from anterior pelvic sway and posterior trunk shift
	Pain exacerbated by movement/postures involving passive lumbar extension
	Pain provoking activities tend to include extension-based passively adopted postures (e.g. sway standing, walking)
	Pain eased by movement/postures involving lumbar flexion (e.g. bending forwards)

### 2.3 Kinematic assessment of posture task performance

Thoracic, lumbar and pelvic kinematics during sitting and standing repositioning tasks were measured using Vicon 512 three-dimensional kinematics motion capture system (Vicon Motion System Ltd. Oxford, U.K.) and a Spinal Wheel (SW); a hand-held spinning wheel device with a retro-reflective marker in its centre, guided along the spinal groove to trace the shape of the spinal curvature. This method was demonstrated to have excellent within-day reliability (intraclass correlation coefficient (ICC) of 0.947–0.980) and high between-day reliability (ICC of 0.719–0.908) with acceptable measurement errors ranging between 1.8 and 4.7° (Sheeran et al., 2010). The spinous process of the 7th cervical (C7), the 12th thoracic (T12) and 1st sacral (S1) vertebrae were identified with retro-reflective markers fixed to the overlying skin in a relaxed sitting position to minimise skin movement (Kuo et al., 2008). Markers were also affixed to the bony landmarks on the pelvis left and right anterior superior iliac spine (ASIS) and posterior superior iliac spine (PSIS). Participants were asked to reproduce a target position defined as a mid-point between end-range spinal flexion and extension. In sitting, participants sat on a fixed height stool with no back rest to minimise haptic cues, moved through the full spinal flexion and extension prior to assuming a mid-point posture between the two extremes and being instructed to memorise this as a target. The target repositioning was performed 4 times with 5 s of relaxed sitting between each repetition. This process was then repeated in standing. The participants were blindfolded for the duration of the testing to minimise any visual cues. No prior training was provided.

### 2.4 Data processing

In Matlab (R2014b), the spinal curvature obtained using the Spinal Wheel was subdivided into 19 equidistant points, and an angle between the lines interconnecting the adjacent points was calculated. Positive values indicated flexion and negative values represented extension. The sum of 12 angles between C7 and T12 and 6 angles between T12 and S1 represented the thoracic and lumbar curvatures, respectively. The pelvic angle was expressed as a sagittal angle between ASIS and PSIS lines relative to horizontal.

Absolute error (AE) representing error magnitude was calculated by averaging values from the four attempts (ignoring positive/negative sign) and subtracting the resultant value from the target.

$$\mathrm{AE}=\Sigma \;\left(\left[x\right]-T\right)/k$$

Key: Σ sum; [*x*] absolute repositioning score; *T* target score; *k* number of trials.

Constant error (CE) representing error direction (target over- or under-shooting) was calculated by subtracting the mean of the four repositioning trials (taking into account positive/negative sign) from the target angle.

$$\mathrm{CE}=\Sigma \;\left(x-T\right)/k$$

Key: Σ sum; *x* repositioning score; *T* target score; *k* number of trials.

Variable error (VE) representing error consistency was obtained by calculating the root mean square of participant's average CE score from the four repositioning trials.

$$\mathrm{VE}=\surd \Sigma \;{\left(\mathrm{SD}-\mathrm{CE}\right)}^{2}/k$$

Key: Σ sum; *SD* standard deviation; *CE* constant error; *k* number of trials.

### 2.5 Data analysis

Group differences in subjects' characteristics for gender were assessed using chi square test. Differences in age and body mass index (BMI) were assessed using a 1-way analysis of variance.

**Fig. 1:**
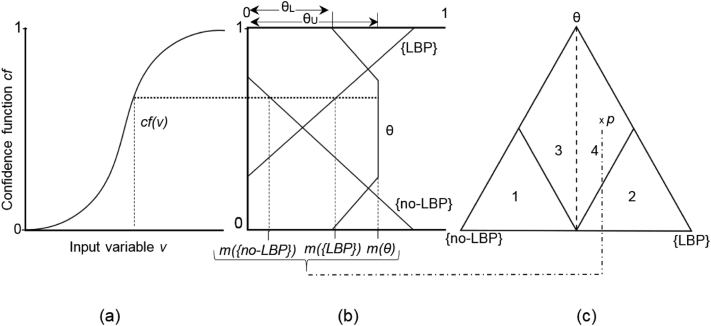
Method of classification illustrating the interaction of 3 main stages. The classification method showing the interaction of its three main stages. (a) Conversion of input variable, v, into confidence factor cf(*v*) using the sigmoid confidence function. (b) Conversion of confidence factor into body of evidence (BOE). Linear relationships between confidence factor and belief functions of pain-free, *m*({no-LBP}, low back pain, *m*{LBP} and uncertainty, m(θ) (solid lines), are constructed using a pre-defined upper (θ_U_) and lower (θ_L_) boundary of uncertainty. The point at which the value of the confidence factor (dotted line) intersects these lines gives the three corresponding belief values (dashed line), *m*({*no-LBP*}), *m*{*LBP*}) and *m*(*θ*), which make up the BOE. (c) The visualisation of the BOE within a simplex plot, denoted by the point *p*. The three belief values are plotted as a distance towards the corresponding vertex {no-LBP}, {LBP} and θ. The simplex plot is divided into four regions: 1 denotes the dominant no-LBP classification region; 2 denotes the dominant LBP classification region; 3 denotes the non-dominant no-LBP classification region and 4 denotes the non-dominant LBP classification regions. The dotted vertical line is the decision boundary between a classification of no-LBP and LBP.

The objective classification was determined using the Cardiff Dempster-Shafer Theory (DST) Classifier previously used to classify locomotor function (Beynon et al., 2006). The DST is a mathematical method that allows data obtained by different means and from different sources to be combined to produce a level of belief (belief function) from the evidence available (Dempster, 1968; Shafer, 1976). The DST method has 2 main functions: Firstly to define an objective non-linear relationship between the magnitude of input variables (e.g. lumbar repositioning error) and a belief the subject belongs to a particular group (e.g. belief the individual has LBP), and secondly to combine these belief values mathematically across numerous input variables to determine a combined body of evidence. This method was described previously to distinguish between the function of individuals with knee and hip osteoarthritis compared to the function of healthy individuals (Beynon et al., 2006; Jones et al., 2006; Whatling et al., 2008) and to determine the relationship between biomechanical and patient-reported outcomes measures following total knee replacement (Biggs et al., 2019; Metcalfe et al., 2017). This study presents a first application of the same approach to classification of LBP, whereby a series of classifications were performed to provide an objective and visual indicator of the LBP sub-types from no-LBP function. The DST classification method is described in detail elsewhere (Beynon et al., 2006, Jones et al., 2006, Whatling et al., 2008). For the current study, classification of LBP from pain-free individuals (no-LBP) is used as an example to describe the 3-stage process: (1) the repositioning error variables were converted into confidence factors to represent a level of confidence in support to a given hypothesis (*x*), (2) confidence factors were converted into a set of belief measures (body of evidences (BoEs)). This two-step process transforms the repositioning error variables from each subject into a set of three belief values: a belief that the subject exhibits function characteristic of LBP, (*m*(LBP)); a belief that the subject exhibits function characteristic of pain-free individuals, (*m*({*LBP*})); and belief in either LBP or no-LBP; (*m*(θ)), in other words level of uncertainty. Multiple variables of sensory function were then used to construct BoEs that offered positive or negative evidence to support the classification. (3) Using the Dempster's rule of combination, a final BoE was constructed consisting of the three belief values with the sum of one. Finally, the BoEs were visualised using simplex plots where each participant's overall function was represented within an equilateral triangle divided into regions providing boundaries for the three belief values (LBP, no-LBP, θ). Participants' function positioned on the left of the central decision boundary has no-LBP function; those on the right have functional characteristics of LBP. The closer the point is situated to a vertex the greater the belief is that the subject has these characteristics (Fig. 1).

In this study, seven sitting and standing repositioning sense function classifiers were created containing measures of (i) LBP-FP and no-LBP (ii) LBP-AEP and no-LBP (iii) LBP-PEP and no-LBP (iv) LBP subgroups combined and no-LBP (v) FP-LBP and AEP-LBP (vi) FP-LBP and PEP-LBP and (vii) AEP-LBP and PEP-LBP. The DST classifier accuracy was assessed using a ‘leave one-out cross validation’ method (Beynon et al., 2006). In this method the classifier control variables are calculated using (*n* − 1) where *n* is the number of subjects within the tested sample. These control variables were used to convert the remaining subject variables to its related *BoE*, whereby classifying the subject. This process was replicated *n* times with ‘leave one-out cross validation’ accuracy calculated as an average of the classification accuracy of all of the ‘left-out’ subjects. The individual ranking of each input variable was then assessed on the whole training body (i.e. all *n* subjects) to determine the most discriminatory variables within each classification.

## 3 Results

### 3.1 Demographics

Out of 224 eligible LBP patients referred for physiotherapy and 30 pain-free controls, 90 (40%) LBP and 28 (93%) pain-free individuals volunteered. Three LBP and two pain-free participants' data were lost leaving 87 LBP (FP = 49, AEP = 23, PEP = 14) and 28 pain-free participants for analysis. The sample demographics are presented in Table 3. Pain and disability scores were similar across the LBP subgroups with pain between 4.6 and 4.9 out of 10 on the visual analogue scale (VAS) and LBP-related disability between 6.0 and 7.3 indicating moderate disability. Gender split varied across LBP subsets with AEP and PEP predominantly consisting of females (Table 3).

**Table 3:** Sample demographics.

Variable	No-LBP	LBP		
		FP	AEP	PEP
	n = 28	n = 49	n = 23	n = 14
	Mean (SD)	Mean (SD)	Mean (SD)	Mean (SD)
Age	35.2 (9.7)	33.3 (10.1)	39.7 (12.9)	33.4 (8.3)
BMI	24.8 (2.2)	25.2 (3.7)	25.0 (3.7)	25.0 (4.5)
VAS	–	4.9 (1.4)	4.6 (1.5)	4.6 (1.5)
RMDQ	–	7.3 (3.8)	6.0 (2.8)	7.1 (4.7)
Gender % Male-female	40%-60%	43%-57%	35%-65%⁎	8%-92%⁎

Key: LBP = low back pain; FP = flexion pattern; AEP = active extension patter; PEP = passive extension pattern; BMI = body mass index; VAS = visual analogue scale; RMDQ = Roland Morris Disability Questionnaire.

^⁎^ Significant at *p* < 0.05.

### 3.2 Repositioning sense in LBP and no-LBP

Thoracic, lumbar and pelvic repositioning error (RE) magnitude (AE), variability (VE) and direction (CE) during sitting and standing tasks were higher in LBP compared to the no-LBP group (Table 4). Whilst there was generally no difference between the LBP subgroups in the error magnitude (AE) and variability (VE), subgroup differences were detected in the error direction (CE). In sitting, the FP group underestimated the target in the lumbar spine and overestimated the thoracic target, whilst in AEP and PEP this pattern was reversed, overestimating lumbar and underestimating the thoracic target. In standing, all three LBP subgroups overestimated the lumbar and underestimated the thoracic target with the size of the error being more prominent in the AEP and PEP. In the pelvis, all three LBP subgroups overestimated the target, with a pelvis tilted anteriorly as a result compared to no-LBP (Table 4).

**Table 4:** Thoracic, lumbar and pelvic repositioning errors means and standard deviation during sitting and standing in LBP and no-LBP.

Position	Region	Variable	No-LBP	LBP		
				FP	AEP	PEP
			n = 28	n = 49	n = 23	n = 14
			Mean (SD)	Mean (SD)	Mean (SD)	Mean (SD)
Sitting	Thoracic	AE	2.8 (1.7)	5.5 (3.7)	4.9 (3.1)	7.8 (5.0)
		VE	2.3 (1.6)	5.1 (1.6)	5.3 (1.7)	5.5 (1.6)
		CE	0.5 (2.3)	−3.2 (4.9)	4.7 (3.1)	7.5 (4.8)
	Lumbar	AE	1.8 (0.7)	7.7 (4.1)	5.7 (2.3)	10.6 (4.8)
		VE	1.9 (0.9)	4.8 (3.1)	3.8 (1.8)	3.7 (1.8)
		CE	0.2 (1.0)	3.2 (6.8)	−3.1 (4.1)	−8.4 (7.9)
	Pelvis	AE	1.8 (1.3)	4.0 (3.0)	3.8 (2.6)	5.1 (1.3)
		VE	1.2 (0.6)	3.5 (1.5)	1.1 (0.6)	1.3 (1.0)
		CE	0.4 (1.9)	−3.1 (3.8)	−2.6 (3.5)	−3.1 (2.7)
Standing	Thoracic	AE	2.6 (1.9)	5.3 (3.3)	5.6 (2.1)	5.8 (3.0)
		VE	1.9 (1.2)	4.4 (2.7)	4.0 (1.9)	4.5 (1.9)
		CE	−0.2 (2.8)	0.8 (4.5)	2.2 (4.1)	1.4 (5.2)
	Lumbar	AE	1.8 (1.3)	6.3 (3.8)	6.2 (2.3)	7.2 (5.6)
		VE	1.8 (1.3)	4.4 (3.0)	5.4 (4.8)	7.1 (5.8)
		CE	−0.3 (0.9)	−1.5 (5.8)	−4.7 (2.7)	−4.8 (2.3)
	Pelvis	AE	0.8 (0.6)	2.2 (1.5)	2.4 (1.3)	1.8 (1.0)
		VE	0.6 (0.6)	0.6 (0.3)	2.7 (1.2)	3.3 (1.0)
		CE	0.3 (0.9)	−1.6 (1.7)	−2.9 (1.3)	−1.3 (1.0)

Key: LBP = low back pain; FP = flexion pattern; AEP = active extension patter; PEP = passive extension pattern; AE = absolute error; VE = variable error; CE = constant error. Negative error indicates extension direction, positive value indicates flexion direction.

### 3.3 Classification findings

**Fig. 2:**
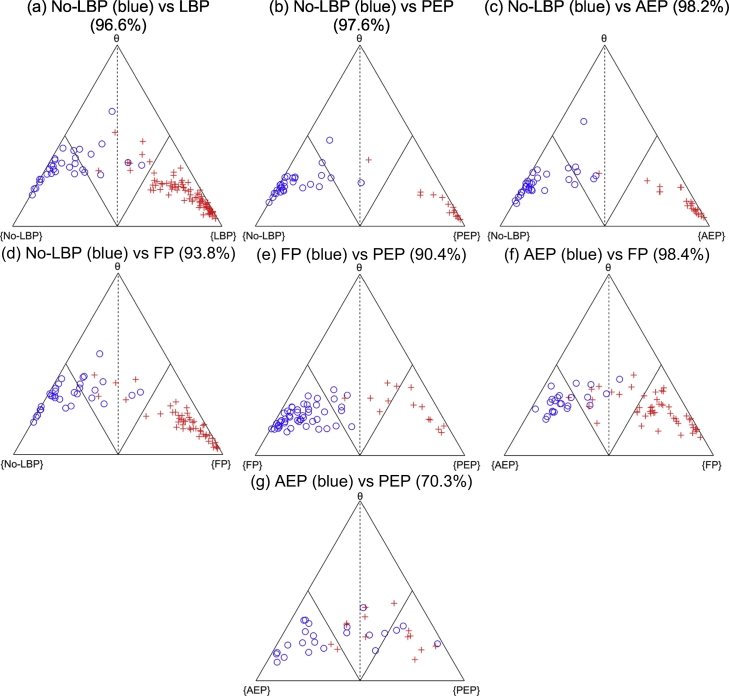
(a–g) DST Simplex plots of the repositioning errors. LBP (low back pain), no-LBP (pain-free), FP (flexion pattern), AEP (active extension pattern), PEP (passive extension pattern).

The baseline classifier was created from the repositioning sense data of the no-LBP individuals. The Cardiff DST method classified LBP (pooled subgroups) from no-LBP with 96.61% accuracy, with 7 out of 87 LBP cases (8%) classified in non-dominant regions indicating a higher level of uncertainty. None of the LBP cases were misclassified as dominant healthy (Fig. 2a). When classifying LBP subgroups from no-LBP, the Cardiff DST method classified FP with an accuracy of 93.83% (Fig. 2b), AEP with an accuracy of 98.15% (Fig. 2c) and PEP with an accuracy of 97.62% (Fig. 2d). 5 out of 45 (11%) of FP, 1 out of 24 (4.1%) AEP and 1 out of 14 (7.1%) PEP cases were classified as uncertain with no cases mis-classified within the dominant healthy region.

Discrimination between the LBP subgroups was more variable. FP and PEP were discriminated with high accuracy of 98.44%, where 6 out of 49 (12%) FP and 6 out of 24 (25%) PEP individuals were classified in non-dominant regions and none were misclassified in dominant regions (Fig. 2e). FP and AEP were classified with 90.41% accuracy with 9 out of 49 (18%) of FP, and 6 out of 24 (25%) AEP subjects classified in non-dominant regions. One (2%) FP case was misclassified as dominant AEP (Fig. 2f). Lower level of discrimination accuracy of 70.27% was detected between AEP and PEP subgroups with 2 out of 14 (14%) in PEP misclassified as AEP and 4 out of 24 (16%) AEP misclassified as PEP (Fig. 2g).

All measured variables were ranked on the basis of the discrimination accuracy percentage (Table 5). When discerning LBP (subgroups pooled) from no-LBP, lumbar AE during sitting and standing were the top two discriminators (92.38% and 86.44%, respectively). When discerning LBP subsets from no-LBP, the most accurate discriminator for FP was lumbar AE during sitting (90.12%) and for AEP it was the lumbar CE during standing (94.44%). For PEP there were three joint top discriminators all at 95.24%; lumbar AE, lumbar CE and pelvic VE during standing (Table 4). Finally, discriminating between the LBP subgroups, pelvic VE during standing was the most accurate discriminator for discerning FP from PEP and FP from AEP with accuracy of 96.88% and 87.67%, respectively. Lumbar CE during sitting was the main discriminator between AEP and PEP with the lowest level of discrimination accuracy of 75.68% (Table 5).

**Table 5:**
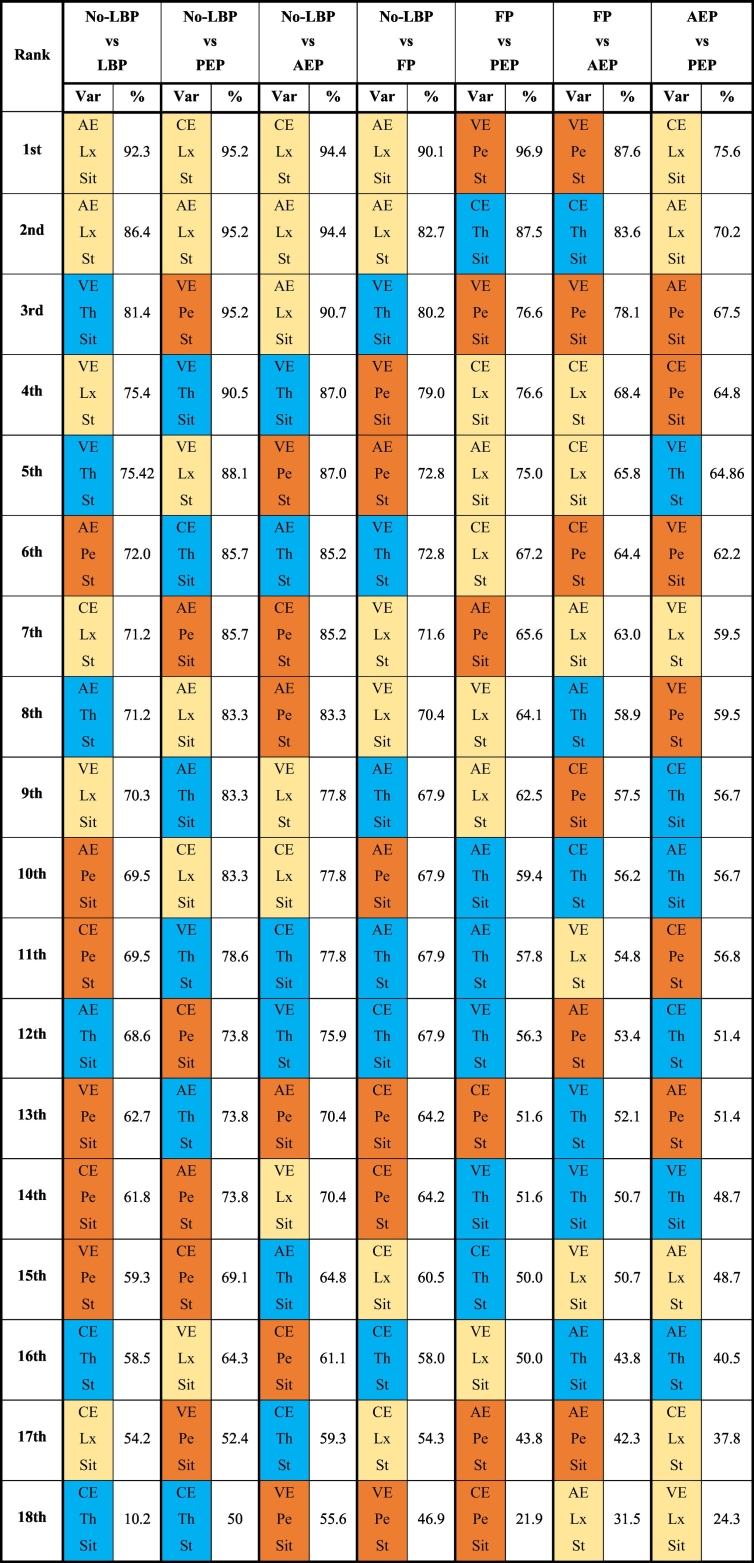
Repositioning sense error measures ranked by percentage level of discriminative accuracy.

Key: LBP = low back pain; FP = flexion pattern; AEP = active extension pattern; PEP = passive extension pattern; Var = variable; AE = absolute error (magnitude); VE = variable error (consistency); CE = constant error (direction); Th = thoracic; Lx = lumbar; Pe = pelvis; Sit = sitting; St = standing.

## 4 Discussion

This study employed a DST Classifier, an objective classification method, to discern between clinical subsets of LBP based on objective measures of repositioning sense in the spine and pelvis during sitting and standing. This is the first time the Cardiff DST Classifier has been used to discern between subsets of a single condition. This is of particular importance in LBP, the heterogeneity of which is recognised as an important factor attributing to low treatment success (Foster et al., 2013).

This study had three main findings: First, within a sample of 115 participants, the Cardiff DST Classifier discriminated no-LBP from LBP (pooled and in subsets) with accuracy ranging between 93.83% and 98.15% with no mis-classified individuals falling within the dominant region of the simplex plot. Second, discrimination between the LBP subsets was more variable with the highest accuracy of 96.8% discriminating LBP flexion from the passive extension subset, 87.7% accuracy discriminating flexion from active extension and 70.27% accuracy discriminating the two extension subsets. Third, the ranking analysis revealed lumbar AE in sitting as the principal variable to discriminate LBP from no-LBP and flexion from no-LBP, whilst lumbar CE in standing most accurately discriminated the LBP extension subsets from no-LBP.

The level of LBP classification accuracy reached by the Cardiff DST Classifier is of significant clinical importance. Clinical classification of LBP involves a complex synthesis of a large amount of subjective and objective information upon which a clinical judgement is made about the LBP subtype. A substantial amount of clinical training is required to reliably classify LBP which is deemed a barrier for successful implementation within the Health Service (Sheeran et al., 2015). The 90%+ classification accuracy reached by the DST Classifier in this study exceeds inter-examiner agreement levels of expert clinicians undergoing >100 h of LBP classification training with Kappa of 0.82 (Fersum et al., 2009). Even the lowest Cardiff DST Classifier accuracy of 70.27% when discerning between the LBP active and passive extension subsets (pain provoked by extension related activities, prolonged standing/walking) is higher than agreement between practitioners undergoing up to 100 classification training hours (Kappa of 0.66) (Fersum et al., 2009). This demonstrates that repositioning sense function analysed with the DST Classifier can identify LBP subsets with accuracy comparable with (and exceeding) that of a clinical expert, with a potential to be of significant clinical value assisting in classification to help practitioners to design and deliver individualised exercise therapies.

Assessment of motor function forms an important part of the clinical classification process (O'Sullivan, 2006). This was corroborated by biomechanical investigations identifying that compared to no-LBP, flexion pattern individuals sit and move nearer the end-range of lumbar flexion whilst extension pattern individuals tend to operate with their lumbar spine in relative extension (Astfalck et al., 2010; Dankaerts et al., 2006a; Hemming et al., 2018; O'Sullivan et al., 2013; Sheeran et al., 2012). These findings were born out of generating a significant amount of spinal-pelvic biomechanical data including proprioception measures and bilateral trunk muscle electromyography during different function tasks. This study demonstrated that the DST Classifier can identify LBP subsets on the basis of performing a repositioning test. This may have clinical implications potentially focussing the LBP assessment on accurate evaluation of proprioceptive function.

The DST Classifier also ranked the repositioning sense variables by their discriminatory power, identifying the lumbar AE and CE as the most powerful variables when discern LBP function from no-LBP. This is in agreement with previous research where both the lumbar AE and CE were greater in LBP compared to pain-free controls (O'Sullivan et al., 2013). Identifying variables that best characterise function (and dysfunction) within each subset may serve as a therapy target and means of evaluating the impact of therapies on clinically important function outcomes.

Interestingly, the nature of repositioning sense deficits reflected the LBP subsets pain presentation pattern. For example, the highest ranking RE in AEP and PEP was in standing, principally reported as most pain provoking posture in these subsets, whilst in FP the highest ranking RE was in sitting (Table 5) typically the most pain provoking posture in FP. This may indicate that the DST method could be sensitive to clinically important parameters ranking them as higher discriminators. This may have implications for establishing therapy and monitoring focus when evaluating the intervention effect in clinical environment.

The 70.27% discrimination accuracy between the two LBP extension subsets (AEP and PEP) was relatively low compared to the other comparisons made. Although the two subsets are distinct from each other in that AEP patients tend to actively adopt hyper-lordotic posture whilst PEP patients tend to passively sway forward into extension, both subsets report extension tasks (e.g. prolonged standing) a principal pain provoking posture and flexion tasks (e.g. bending forwards) pain easing. This clinical similarity in pain presentation may reflect similarity of the repositioning sense deficits resulting in Cardiff DST Classifier unable to distinguish the difference between PEP and AEP in nearly 30% of cases. This AEP and PEP subset similarity was demonstrated previously in repositioning sense and muscle activity (Sheeran et al., 2012), potentially indicating that despite the two subsets appearing to be different clinical entities, the exercise approach may not need to differ substantially.

The main strength of this study is the sample size (total of 115 participants), more than double the number of participants used in previous Cardiff DST Classifier studies (Beynon et al., 2006; Whatling et al., 2008; Worsley et al., 2016). This sample size allowed establishing STV ratio in region of 10:1 which significantly improved the predictive ability of the DST analysis and allowed for the first to date single condition subset analysis.

The limitation is in the repositioning sense data being obtained using a laboratory based 3-D Vicon motion capture system potentially reducing the clinical applicability. Nevertheless, there are rapid developments of portable motion analysis devices which can be utilised in clinical setting (Al-Amri et al., 2018; Sheeran et al., 2018). Such devices are capable of harnessing volumes of high-quality biomechanical data in the clinical setting. This study provides an important step towards using well-designed objective classification methods to analyse the biomechanical evidence that could be obtained from such portable devices in the clinical setting. Further research is required however, utilising portable devices to obtain data and longitudinal assessment to evaluate the stability of the measures over time as well as the classifier's ability to track any changes in function in response to intervention or diseases progression.

Regarding clinical implications, LBP is considered a highly heterogeneous with a targeted approach to management long advocated as being essential to improve treatment efficacy (Rabey et al., 2019). This study demonstrated that the DST objective classification method can i) accurately distinguish between different subtypes of LBP on the basis of biomechanical measures of proprioceptive function and ii) rank the variables by discriminatory power. Such information can be used to personalise exercise and rehabilitation protocols for each individual patient. For example, an exercise protocol for LBP patient, classified by the Cardiff DST Classifier as flexion pattern with lumbar proprioceptive deficit in sitting ranked the highest discriminator, may focus on lumbar proprioceptive training in sitting.

## 5 Conclusion

The Cardiff DST Classifier utilised biomechanical evidence of spinal and pelvic proprioceptive function to successfully distinguish clinically recognised subsets of LBP from healthy controls. This is a first to date example of discerning between clinically important subtypes of a single condition, achieving a classification accuracy comparable to that of an expert clinician. The repositioning sense variables that best characterise the studied LBP subsets were also identified providing a potential target for management. This study demonstrated the potential of the DST method to simplify the classification process of complex health conditions such as LBP, assisting practitioners to better target treatments with a clear focus on clinically important outcomes for each individual.
